# Supplementary material for: SARS-CoV-2 seroprevalence in a high-altitude setting in Peru: adult population-based cross-sectional study
Source: PeerJ. 2021 Sep 20;9:e12149. doi: 10.7717/peerj.12149 (PMC8459728; doi:10.7717/peerj.12149)
Supplement: Supplemental Information 2 [file peerj-09-12149-s002.docx]

**>> ENGLISH VERSION - QUICK TRANSLATION <<**

**Study title:** Incidence of COVID-19 infection and propagation factors in a cohort of high-altitude residents (Cusco, 3300 masl)

**INDIVIDUAL QUESTIONNAIRE**

**INSTRUCTIONS:** To be filled out by the participants. The forms will be collected at the end of the sampling process

**FAMILY CODE** ________________ (This is on the interviewer's file)

DNI: ____________________

- - - 1. Degree of instruction (Mark only the highest degree obtained)
- Basic or junior: 1. Finished ( ) 0. Discontinued ( )
- High school 3. Finished ( ) 2. Discontinued ( )
- Technique. 5. Finished ( ) 4. Discontinued ( )
- Universitary 7. Finished ( ) 6. Discontinued ( )
- Without instruction: 9 ( )
  - - 1. ¿ How long have you lived at your home? ____________________
      2. ¿ Are you made trips outside the city in the last five months?

NOT ( ) YET ( ) 🡪 ¿Where did you travel? ________________________

- - - 1. ¿ What is your usual job?: ____________________________________
      2. In the last week, how many average hours are you away from home per day? [___] HOURS PER DAY
      3. The quarantine in Peru lasted 107 days. How many approximately did you do? [_____] DAYS
      4. ¿ Has a doctor told you what you have…? (Mark with an X if YES)

HBP ( ) Cancer ( ) ¿Which? _______________________

DM ( ) Other cardiac disease ( ) ¿Which? ___________

Renal disease ( ) Asthma ( )

- - - 1. Do you take medication daily? NOT ( ) YES ( ) 🡪 ¿Which one? __________________________
      2. Approximately, how much is your WEIGHT: _____Kg HEIGHT: _____meters
      3. How many people do you have daily contact with?[_____] PEOPLE
      4. How many times do you leave the house a week?[_____] DAYS A WEEK
      5. During the past 3 months, have you had any of the following symptoms? (mark with X)

| Cough |  | Fever / chills |  | Headache |  |
| --- | --- | --- | --- | --- | --- |
| Throat pain |  | General discomfort |  | Muscle pain |  |
| Back pain |  | Diarrhea |  | Decreased smell / taste |  |
| Difficulty breathing |  | Nasal congestion |  | Others:________________  _____________________ | |

- - - 1. What are the protection measures that you apply? **(mark with X)**

|  | Never | Sometimes | Frequently | Always |
| --- | --- | --- | --- | --- |
| I am more than 1 meter away from other people |  |  |  |  |
| Use of mask |  |  |  |  |
| Use of N95 mask |  |  |  |  |
| Use of a face shield |  |  |  |  |
| Use of gloves |  |  |  |  |
| Use of alcohol to desinfect your hands |  |  |  |  |
| I wash my hands constantly |  |  |  |  |

- - - 1. During the pandemic; have you had any diagnostic tests for COVID-19?

**NOT ( ) YES ( )**🡪 **¿How many?** [_____]

- - - 1. ¿ Have you had a positive result for COVID?

**NOT ( ) YES ( )** 🡪 **¿Which? _____________________/ ¿When? _____________________**

**Título del estudio:** Incidencia de infección por COVID-19 y factores de propagación en una cohorte de pobladores residentes a gran altitud (Cusco, 3300msnm)

**FICHA DE RECOLECCIÓN INDIVIDUAL**

**INSTRUCCIONES: Para ser llenada por los participantes. Se recolectarán las fichas al final del proceso de toma de muestra.**

**CÓDIGO DE FAMILIA ________________ (Figura en la ficha que tiene el entrevistador)**

DNI: ____________________

- - - 1. Grado de instrucción (Marcar solo el mayor grado obtenido)
- Primaria: 1. Completa ( ) 0. Incompleta ( )
- Secundaria 3. Completa ( ) 2. Incompleta ( )
- Superior técnica. 5. Completa ( ) 4. Incompleta ( )
- Superior universitaria 7. Completa ( ) 6. Incompleta ( )
- Sin instrucción: 9 ( )
  - - 1. ¿Desde hace cuánto tiempo vive en su domicilio? ____________________
      2. ¿Realizó viajes fuera de la provincia los últimos cinco meses?

NO ( ) SI ( ) 🡪 ¿A dónde viajó? ________________________

- - - 1. ¿Cuál es su trabajo habitual?: ____________________________________
      2. La última semana ¿Cuántas horas promedio está fuera de casa al día?[___] HORAS AL DÍA
      3. La cuarentena en el Perú duró 107 días ¿Cuántos aproximadamente hizo usted? [_____] DÍAS
      4. ¿Algún médico le ha dicho que tiene? (Marque con una X si es SÍ)

Hipertensión Arterial ( ) Cáncer ( ) ¿Cuál? _______________________

Diabetes ( ) Otra enfermedad cardiaca ( ) ¿Cuál? ___________

Enfermedad Renal ( ) Asma ( )

- - - 1. ¿Toma medicación diariamente? NO ( ) SI ( ) 🡪 ¿Cuál? __________________________
      2. Aproximadamente, cuánto es su PESO: _____Kg TALLA: _____metros
      3. ¿Con cuántas personas tiene contacto diariamente?[_____] PERSONAS
      4. ¿Cuántas veces sale de casa a la semana?[_____] DÍAS A LA SEMANA
      5. Durante los últimos 3 meses ¿ha tenido alguno de los siguientes síntomas? **(marque con X)**

| Tos |  | Fiebre/escalofríos |  | Dolor de cabeza |  |
| --- | --- | --- | --- | --- | --- |
| Dolor de garganta |  | Malestar general |  | Dolor muscular |  |
| Dolor de espalda |  | Diarrea |  | Disminución del olfato/gusto |  |
| Dificultad para respirar |  | Congestión nasal (moquillo) |  | Otros:________________  _____________________ | |

- - - 1. ¿Cuáles son las medidas de protección que usted aplica? **(marque con X)**

|  | Nunca | A veces | Frecuentemente | Siempre |
| --- | --- | --- | --- | --- |
| Me alejo más de 1 metro de la gente |  |  |  |  |
| Uso de mascarilla |  |  |  |  |
| Uso de máscara N95 |  |  |  |  |
| Uso de careta o protector facial |  |  |  |  |
| Uso de guantes |  |  |  |  |
| Uso de alcohol para desinfectante las manos |  |  |  |  |
| Me lavo las manos constantemente |  |  |  |  |

- - - 1. Durante la pandemia. ¿Se ha realizado alguna prueba para diagnóstico de COVID-19?

**NO ( ) SI ( )**🡪 **¿Cuántas veces?** [_____]

- - - 1. ¿Ha tenido algún resultado positivo para COVID?

**NO ( ) SI ( )** 🡪 **¿Cuál? _____________________/ ¿Cuándo? _____________________**
