# Supplementary material for: SARS-CoV-2 seroprevalence in a high-altitude setting in Peru: adult population-based cross-sectional study
Source: PeerJ. 2021 Sep 20;9:e12149. doi: 10.7717/peerj.12149 (PMC8459728; doi:10.7717/peerj.12149)
Supplement: Supplemental Information 3 [file peerj-09-12149-s003.docx]

**Table supplementary:** Extended analysis of Table 2 of the article

**Title:** Prevalence of serum antibodies to SARS-CoV-2 in the general population at different altitudes in Cusco, Peru, according to individual/family characteristics

|  | Seropositive to SARS-CoV-2 antibodies | | Crude PR | Adjusted PR |
| --- | --- | --- | --- | --- |
|  | N | % |  |  |
| *Global prevalence* |  | 33·1% (30·1 - 36·4%) |  |  |
| *Gender* |  |  |  |  |
| Male  Female | 256  380 | 31·1% (27·3 -35·2%)  34·6% (31·2 -38·3%) | 1  1.12 (0.99-1.26) | 1  1.09 (0.97-1.24) |
| *Study setting* |  |  |  |  |
| Cusco city  Periphery of Cusco  Quillabamba | 248  306  83 | 38·8% (33·4 -44·9%)  34·9% (30·4 -40·1%)  20·3% (16·2 -25·6%) | 1.90 (1.44-2.50)  1.72 (1.31-2.25)  1 | 1.85 (1.41-2.43)  1.71 (1.31-2.22)  1 |
| *Age group (years)* |  |  |  |  |
| >=18, <40  40-59  > 60 | 305  251  76 | 34·8% (31·0 -39·1%)  35·8% (31·8 -40·2%)  24·7% (19·7 -30·9%) | 1  1.02 (0.89-1.17)  0.71 (0.57-0.89) | 1  0.99 (0.86-1.14)  0.65 (0.51-0.83) |
| *Education level (years)* |  |  |  |  |
| <7  7 -11  > 12 | 69  213  340 | 34·2% (26·9-43·3%)  39·2% (34·4-44·7%)  30·3% (26·9-34·0%) | 1  1.15 (0.89-1.48)  0.89 (0.69-1.14) | 1  1.09 (0.86-1.40)  0.79 (0.61-1.03) |
| *BMI* |  |  |  |  |
| Normal  Overweight  Obese | 253  227  118 | 34·1% (30·0-38·6%)  31·1% (27·1-35·7%)  35·5% (30·2-41·8%) | 1  0.91 (0.78-1.07)  1.04 (0.86-1.26) | 1  0.95 (0.81-1.13)  1.17 (0.95-1.44) |
| *Number of people living together* |  |  |  |  |
| 1 - 2  3 - 5  6 -10  > 11 | 34  269  227  77 | 20·5% (14·4-29·1%)  31·8% (27·6-36·7%)  37·5% (31·9-44·0%)  38·7% (28·1-53·2%) | 1  1.55 (1.07-2.27)  1.83 (1.25-2.69)  1.89 (1.18-3.02) | 1  1.47 (1.01-2.15)  1.68 (1.14-2.46)  1.71 (1.07-2.73) |
| *Self-report of COVID-19 case at home* |  |  |  |  |
| No  Yes | 348  272 | 26·2% (22·9-29·9%)  50·2% (44·4-56·7%) | 1  1.91 (1.60-2.29) | 1  1.90 (1.60-2.27) |
| *Death at home (any cause)* |  |  |  |  |
| No  Yes | 551  65 | 31·9% (28·7-35·3%)  50·8% (38·4-67·2%) | 1  1.59 (1.18-2.14) | 1  1.50 (1.11-2.04) |
| *Previous SARS-COV-2 test (rapid test or molecular)* |  |  |  |  |
| No  Yes | 330  303 | 28·7% (25·3-32·6%)  39·9% (35·7-44·7%) | 1  1·39 (1·20-1.62) | 1  1·46 (1·26-1.69) |

Models adjusted for age, gender, and study setting.
